# Mechanical Properties of Hybrid Carbonized Plant Fibers Reinforced Bio-Based Epoxy Laminates

**DOI:** 10.3390/polym13193435

**Published:** 2021-10-07

**Authors:** Edgar Adrián Franco-Urquiza, Raúl Samir Saleme-Osornio, Rodrigo Ramírez-Aguilar

**Affiliations:** 1 Center for Engineering and Industrial Development, National Council for Science and Technology (CONACYT—CIDESI), Querétaro 76265, Mexico; 2 Center for Engineering and Industrial Development (CIDESI), Querétaro 76265, Mexico; raul.saleme@cidesi.edu.mx (R.S.S.-O.); rodrigo.ramirez@cidesi.edu.mx (R.R.-A.)

**Keywords:** natural fiber, henequen fiber, ixtle fiber, thermal shock, viscoelastic properties, bio-epoxy composites

## Abstract

In this work, henequen and ixlte plant fibers were carbonized in a horizontal quartz tube furnace. Several carbonized and non-carbonized fiber fabric configurations were impregnated with a bio-based epoxy resin through the infuseon process. The infrared spectra revealed characteristic bands of styrene instead of organic compounds, representing that the carbonization procedure was adequate to carbonize the plant fibers. The porosity volume ratio for the non-carbonized henequen laminates showed the highest number of voids >1.9%, and the rest of the composites had a similar void density between 1.2–1.7%. The storage modulus of the non-carbonized and carbonized henequen laminates resulted in 2268.5 MPa and 2092.1 MPa, respectively. The storage modulus of the carbonized ixtle laminates was 1541.4 MPa, which is 37.8% higher than the non-carbonized ixtle laminates and 12% higher than henequen composites. The laminates were subject to thermal shock cycling, and tomography scans revealed no alterations on the porosity level or in the cracks after the cycling procedure. Thermal shock cycling promoted the post-curing effect by increasing the glass transition temperature. The viscoelastic results showed a variation in the storage modulus when the carbonized fiber fabrics were located between natural fiber fabrics, which was attributed to more excellent compaction during the infusion process. Variations in the viscoelastic behavior were observed between the different types of natural fibers, which influenced the mechanical properties.

## 1. Introduction

Biolaminates are natural fiber-reinforced bio-based epoxy resins [1,2,3]. Vegetable oils obtained from soybeans and flaxseeds are suitable for producing epoxy groups with high functionality due to their relatively high iodine value and high unsaturated fatty acid content [4,5,6]. Recent evaluations have reported that bio-based epoxy resins synthesized from functionalized natural compounds may have comparable thermal and mechanical properties with commercial epoxy resins [2,4,6,7,8,9,10,11]. The three main components of plant fibers are cellulose, lignin, and hemicellulose, where cellulose is responsible for the inherent strength and stability of the natural fibers, whereas hemicellulose contributes to their structure [12,13,14,15,16,17,18,19]. Plant fibers are renewable, non-toxic, non-abrasive, biodegradable, and environmentally friendly. Furthermore, plant fibers have many advantages over synthetic fibers due to their abundance, low cost, high specific mechanical properties, and density values of 1.2 to 1.6 g/cm^3^, all of which allow the manufacturing of lightweight composites. Thus, they are attractive for various sectors such as the infrastructure, aviation, or automobile industries.

The global cellulose fiber market is primarily driven by the significant demand from the textile industry. The previously mentioned factors reflect that the intrinsic properties and the abundant availability and accessibility of plant fibers are the primary reasons for an emerging new interest in sustainable technology. However, there are not currently any markets that are ready for natural fiber-oriented applications, which is essentially because properties of these natural-fiber based products vary depending on the quality of the harvest, age, and body of the plant from which they are extracted. The absence of markets where plant fibers are in high demand provokes the abundance of residual plant fibers and forces farmers to eliminate the waste fibers by burning crop fields.

Crop residue burning is an inexpensive and effective method to remove excessive plant residue, help timely planting, and control pests and weeds. Moreover, carbonized plant fibers can be used as a reinforcement element in composite laminates.

The high carbon content and an aromatic structure make lignin a suitable candidate for carbonization to produce carbonized fibers. Balint et al. [20] carbonized soy hull fiber at 500 °C and 900 °C to obtain different types of biocarbon that could be used as fillers in a polyamide matrix (PA66). They found a higher number of functional groups when the natural fibers were treated at lower temperatures, which also interrupt the crystallite growth of PA66 due to a solid bond connection, while a lower number of functional groups promotes heterogeneous crystallization. Both biocarbons resulted in an increased storage modulus and glass transition temperature over PA66. Marija Vukcevic et al. [21] used waste hemp fibers as a low-cost precursor to produce carbon-based sorbents for pesticide analysis in water samples. The carbon materials were prepared through the carbonization of unmodified and chemically modified natural fibers, and the sorption properties were discussed. The specific surface area and the oxygen groups increased in the carbonized materials modified with potassium hydroxide, and the results indicated that activated carbon hemp fibers are effective as a solid-phase sorbent for pesticide analysis in water samples. Vida et al. [22] worked on developing carbonized lignin fibers produced by alkali organosolv lignin and electrospun solutions followed by carbonization that consisted of different heating programs for carbonization. They observed that the carbonized fibers had a smooth surface with a regular diameter and that they were controlled by the carbonization process and lignin type. They stated that this kind of material could be oriented to energy storage devices and water or gas purification systems. Mingchao Zhang et al. [23] developed susceptible wearable strain sensors based on carbonized cotton fabrics. The strain sensors exhibited an extensive workable strain range, notorious sensitivity, inconspicuous drift, and long-term stability, simultaneously offering the advantages of low cost and simplicity in terms of device fabrication and versatility in terms of applications for wearable electronics and intelligent robots. Johanna M. Spörl et al. [24] investigated the mechanical properties of cellulose-based carbon fibers via the application of carbonization agents. They used a continuous process at relatively low temperatures and high carbon yields using ammonium dihydrogen phosphate and ammonium tosylate as sulfur-based carbonization agents. The authors observed that both precursors were suitable for continuous processing in terms of mechanical stability, and this resulted in carbonized fibers with a significant increase in the mechanical properties, especially in Young’s modulus. However, they recognized the need for deeper studies in order to evaluate the resulting structural changes.

Our literature review allowed us to find many and extensive original articles that deal with the development of innovative activated carbon adsorbents from natural plant fiber waste, and only a small part of that research discussed the use of carbonized plant fibers as a reinforcement for polymers. However, there is no information regarding the evaluation of the mechanical performance of carbonized plant fiber-reinforced bio-based epoxy resins, which is the gap this work intends to fill.

This work aims to evaluate the mechanical performance of bio-epoxy resin reinforced with natural and carbonized agave fiber fabrics. Of particular interest is in the mechanical properties that are demonstrated when these hybrid bio-laminates are subject to thermal shock cycles, which allows the discovery of multiple industrial applications.

## 2. Materials and Methods

### 2.1. Materials

Henequen and ixtle yarn fibers with thread counts of 35 and 30, respectively, were purchased in Cordeleria Santa Ines, Yucatan, Mexico. The average length of the yarn was 2000 m, and the yarn was rolled on a reel for the following weaving steps. Both natural fibers were woven in a plain weave configuration by Mexican artisans from the state of Queretaro in Mexico. Henequen and ixtle fibers have an elastic modulus of 13 and 27 GPa, a tensile strength of 13 and 18 MPa (ASTM C1557), and a density of 1.12 and 1.02 g/cm^3^ (ASTM D3822), respectively. More information on the mechanical properties of both plant fibers is available in previous work [2].

The plant fibers were impregnated with a bio-based epoxy resin. The epoxidized vegetable oil (EVO) Surf Clear, with a biobased carbon content of approximately 40%, and the SD EVO fast hardener from Sicomin Epoxy Systems^®^ (Provenza-Alpes-Costa Azul, Châteauneuf les Martigues, France) was mixed in a ratio by volume of 2/1. According to the technical datasheet, EVOH has a flexural modulus of 3.2 GPa, bending strength of 117 MPa, and flexural strain of 8.5%. According to the supplier, the EVO was extracted from vegetable oils with no access to their chemical structure, offering an alternative due to the combination of specific attributes such as low viscosity, biodegradability, and high epoxy functionality.

### 2.2. Composite Manufacturing and Testing

The carbonization process of both plant fibers was performed in a horizontal tube furnace HT2-1100 from Prendo using an inert nitrogen atmosphere. The furnace contained a quartz tube with an outside diameter of 60 mm, a 5 mm wall thickness, and was 1000 mm in length. One end was sealed with a stainless-steel gasket and flange, while the other side was used to load/unload the gas inlet/outlet for the carbonization the of plant fibers. The furnace was manufactured with ceramic fiber insulation that allows temperatures up to 1100 °C. It contained one heating zone located in the middle of the quartz tube.

The plant fiber fabrics were cut into 150 × 40 mm rectangular specimens. Each fiber specimen was weighed and placed in the quartz tube, with two specimens being placed per cycle. The air was evacuated from the system by supplying an N_2_ flow of 0.8 L/min through a gas flow controller. The flow was gradually increased to 2.1 L/min to fill the furnace. The carbonization protocol consisted of heating from 20 to 270 °C at 1.5 °C/min with an isothermal step for two hours. It was subsequently heated from 270 to 600 °C at 2 °C/min. At the end of the protocol, the system was cooled down. Once the temperature reached 50 °C, the gas flow was stopped, and the quartz tube was disconnected to prevent it from collecting the calcined fiber specimens, which were placed inside a vacuum desiccator for 14 h. Then, the fibers were left to degas overnight before further processing. It should be noted that the quartz tube needed to be washed after each carbonization cycle.

The hybrid laminates consisted of four layers with the same stacking sequence (0/90°) but different configurations, as presented in Table 1. The non-carbonized fibers were used without any thermal or chemical treatments.

**Figure 1:**
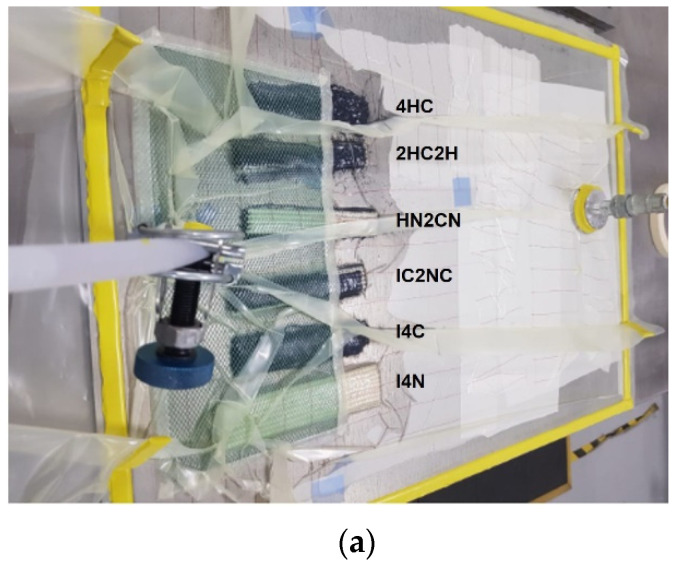

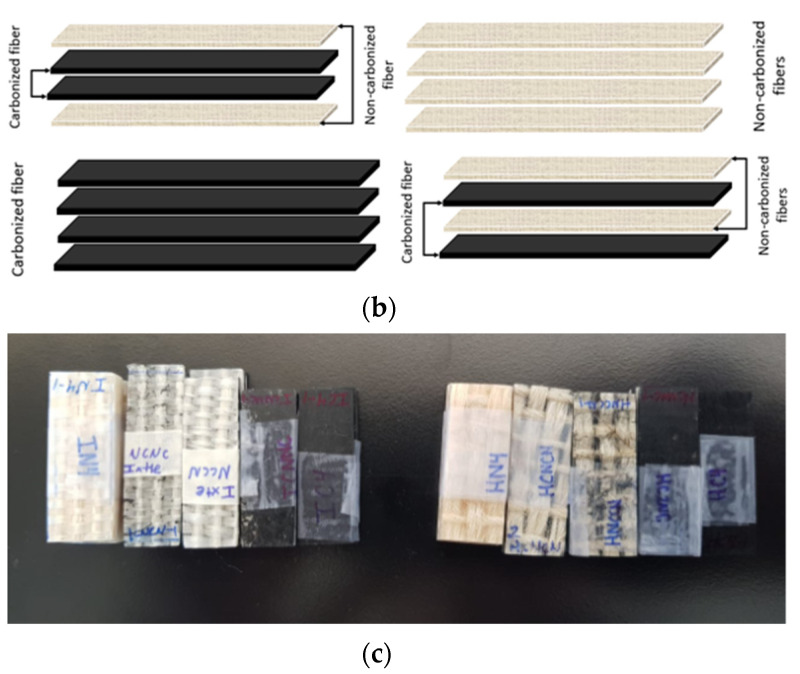
Photographs corresponding to (**a**) VARI process during impregnation of hybrid laminates, (**b**) schematic representation of carbonized composites, (**c**) obtained specimens.

The Vacuum assisted resin infusion (VARI) was used to prepare the laminates. The vacuum pressure was −20 inHg, and the curing reaction lasted for 24 h at 25 °C. Laminates contained four plain weave layers with the same stacking sequence and 150 mm × 40 mm × 3 mm as nominal laminate dimensions. On the top of the substrate, peel ply and distribution mesh were placed over the fiber, and the entire configuration was covered with a vacuum bag and was conducted using sealant tape. After air evacuation, the resin was infused at room temperature. The resin inlet and outlet positions were at the edges of the hybrid laminates, respectively. Figure 1 shows the VARI process during the resin impregnation of hybrid laminates and the obtained specimens.

### 2.3. Characterization

The carbonized and non-carbonized plant fibers were analyzed using Fourier-transform infrared spectroscopy (FTI-IR) Perkin Elmer Frontier with attenuated total reflectance (ATR). The FTIR spectra were recorded by scanning the samples in the frequency range of 500–4000 cm^−1^. The viscoelastic behavior of the laminates was evaluated in a Dynamical Mechanical Analyser TA Instruments DMA Discovery 850, according to the ASTM-D7028. The specimens with the nominal dimensions of 50 mm × 12 mm × 3 mm were tested using the single cantilever geometry to conduct measurements from room temperature to 120 °C with a heating ramp of 5 °C/min. X-ray computed tomography (CT) is a non-destructive technique for visualizing interior features within solid objects and for obtaining digital information on their three-dimensional (3D) geometries and physical properties. The General Electric Phoenix phoenix v|tome|x m is a versatile X-ray microfocus CT system for 3D metrology and analysis with up to 300 kV/500 W. The CT system was used to reveal manufacturing defects and to quantify voids present in the laminates with the rectangular dimensions of 50 mm × 12 mm × 3 mm. The results were analyzed using the Baker Hughes software VGEasyPore algorithm. The thermal shock was conducted in a CM Envirosystems Thermal Shock Chamber model KTS-120-B2V. The DMA specimens with the nominal dimensions 50 mm × 12 mm × 3 mm were exposed to thermal shock cycling, which started from room temperature and then increased to +80 °C over the course of 30 min. After that, the temperature was decreased to -50 °C over the same amount of time. This sequence was repeated for 30 cycles. CT scans were performed before and after DMA tests.

## 3. Results and Discussion

**Figure 2:**
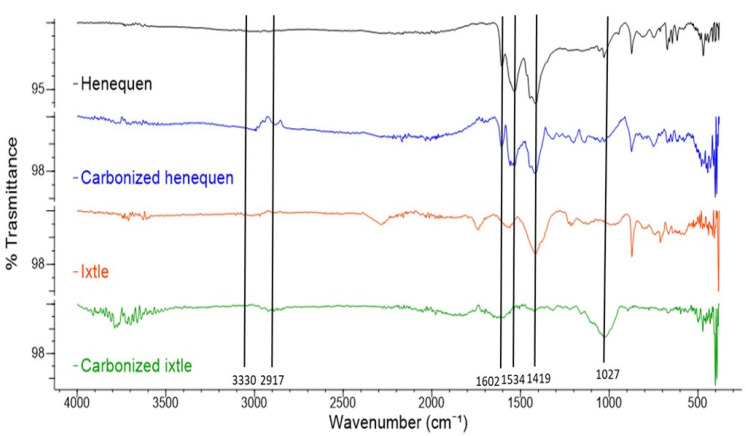
FTIR spectrum of the natural fiber and the carbonized natural fiber.

FTIR allows for the identification of the functional groups and the molecular bond structures with IR spectrum bands. Furthermore, FTIR also indicates effective molecular structure transformations occurring due to physical and chemical treatments. Figure 2 shows the FTIR spectra corresponding to the carbonized and non-carbonized agave fibers.

It is possible to appreciate the similar band positions in the henequen and ixtle fibers that are associated with similar agave fibers, as expected. The broad peak at 3200–3500 cm^−1^ can be attributed to the O–H hydroxyl group stretching vibration in the cellulose molecules. The band identified between 2900 and 3100 cm^−1^ corresponds to the C–H alkyl group stretching vibration in the cellulose, lignin, and hemicellulose aliphatic bonds. According to [25], these two bands represent the macromolecular interactions between cellulose and hemicellulose and the water in the fibers, both of which promote the binding of water molecules to these characteristic hydroxyl groups. The band at around 1740 cm^−1^ can be ascribed to the acetyl and ester groups of the hemicelluloses and the aromatic components of lignin. The band at around 1640 cm^−1^ is related to the O–H bending of the water absorbed into the cellulose fiber structure, and the absorption bands at 1602 cm^−1^ and 1505 cm^−1^ are associated with the C–C in-plane symmetrical stretching vibration aromatic rings that are present in lignin. The pectin constituting the fibers appears at the vibration of the carboxyl bond between 1430 and 1420 cm^−1^. The peak at 1239 cm^−1^ is due to the C-O stretching vibration of the acyl group present in the lignin. The cellulose is shown by the C-O-C bond stretching at the wavelength of 1023 cm^−1^, which is the characteristic of the polysaccharide nature of the fiber [26].

In the case of carbonized fibers, it can be observed that the infrared spectrum shows two characteristic bands at 1534 cm^−1^ and at 1419 cm^−1^, which correspond to N-H and C=C stretching, respectively, and are caused by the elimination of all of the other organic bonds by means of the pyrolysis. The absence of characteristic signals between 3500 and 1700 cm^−1^ confirms the complete carbonization of the plant fibers.

During the vacuum infusion process, there was a creaking sound that could be heard. The creaking sound was attributed to the crushing of the carbonized fibers due to the vacuum pressure. Carbonized fibers have a highly porous structure due to the changes that occur when transforming from an organic to an inorganic structure [27]. The crunch was not heard during the VARI process of non-carbonized agave fabric laminates.

Voids or bubbles can be formed due to incomplete resin filling, air entrapment, or disrupted resin flow caused by the presence of inorganic fillers. These voids considerably deteriorate the mechanical performance of laminates. X-ray CT is becoming increasingly important among non-destructive inspection techniques for FRP composites because it uses material densities to detect and count defects in laminates [28,29]. In this work, X-ray CT was used to perform a porosity analysis on the DMA specimens.

**Figure 3:**
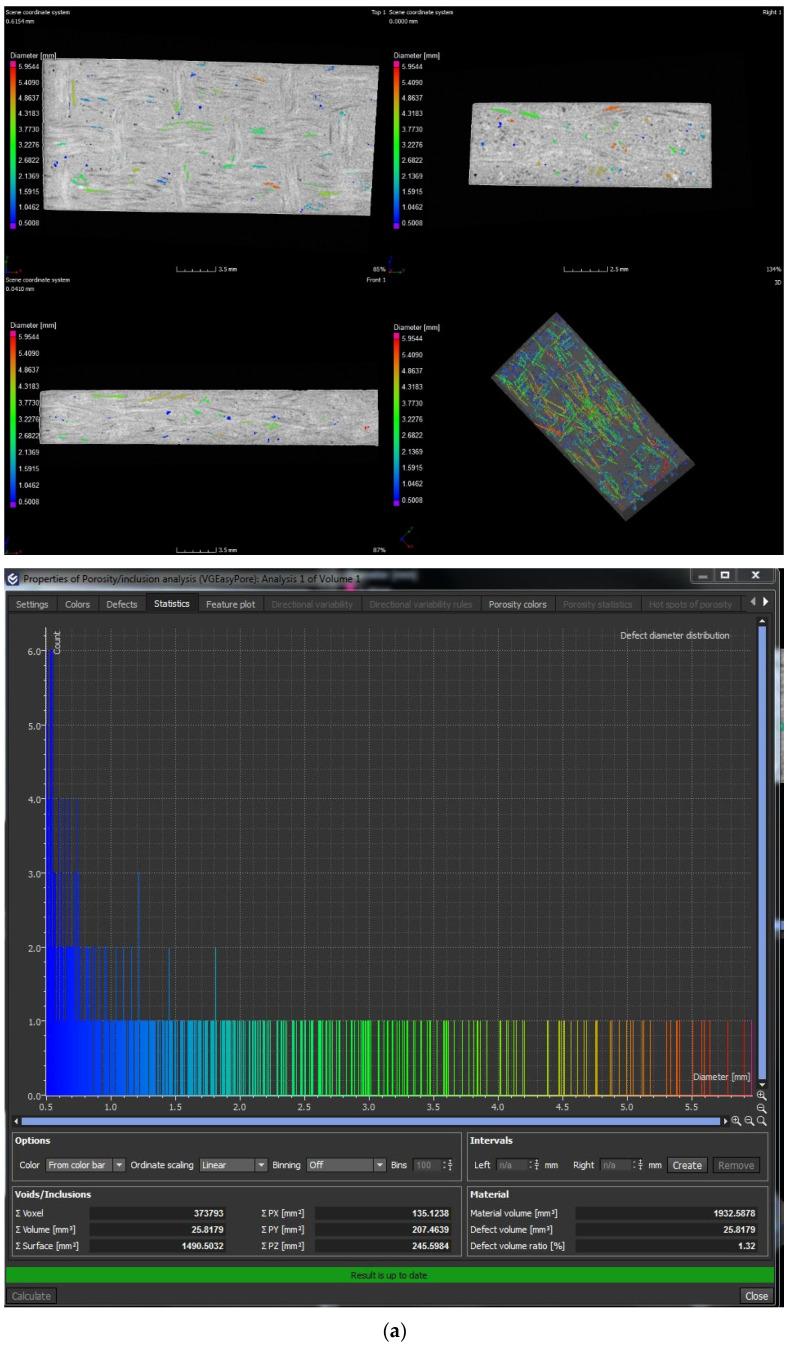

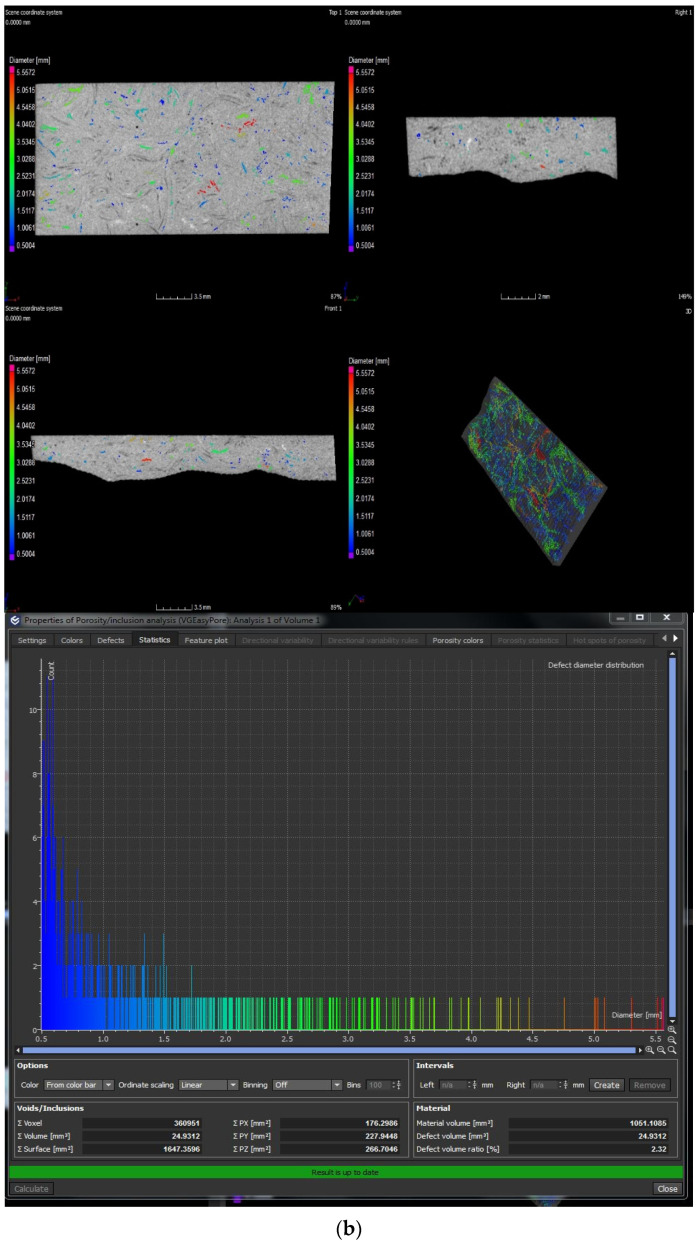

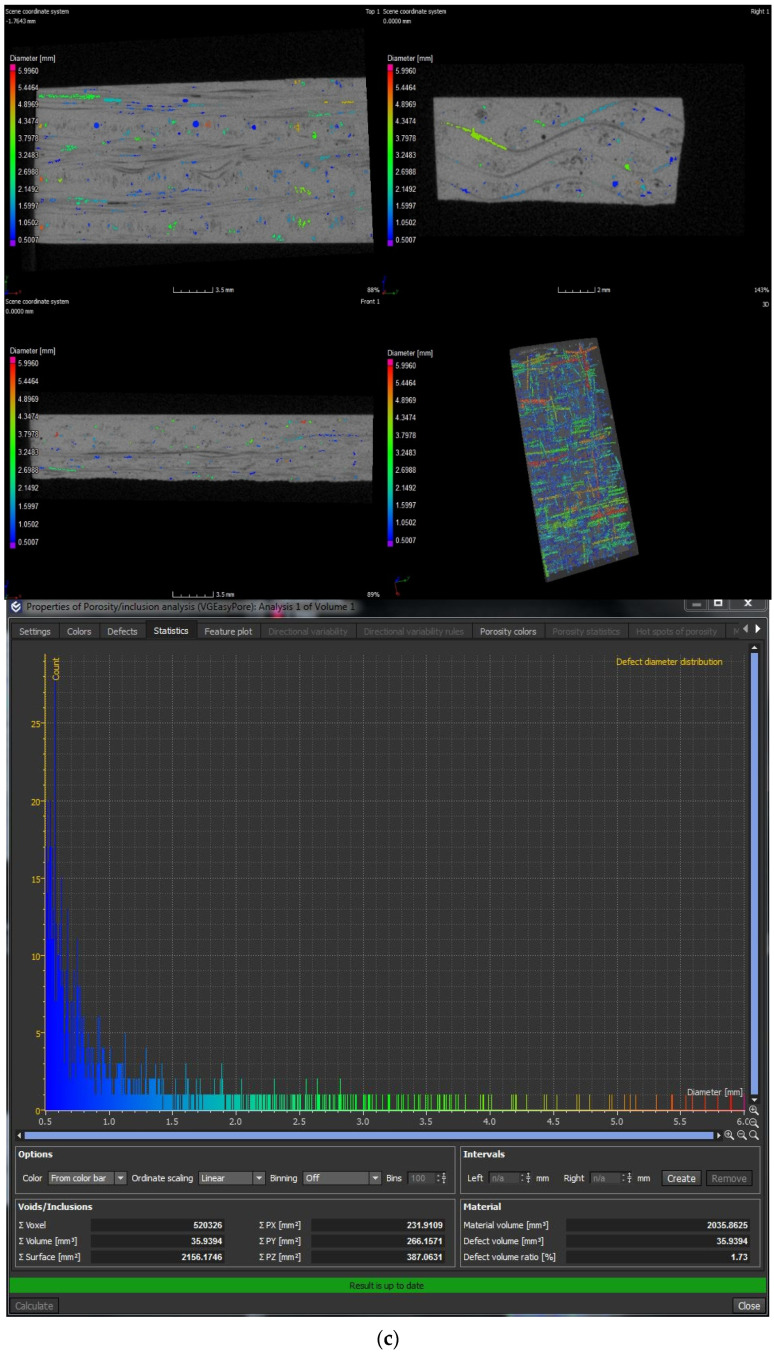

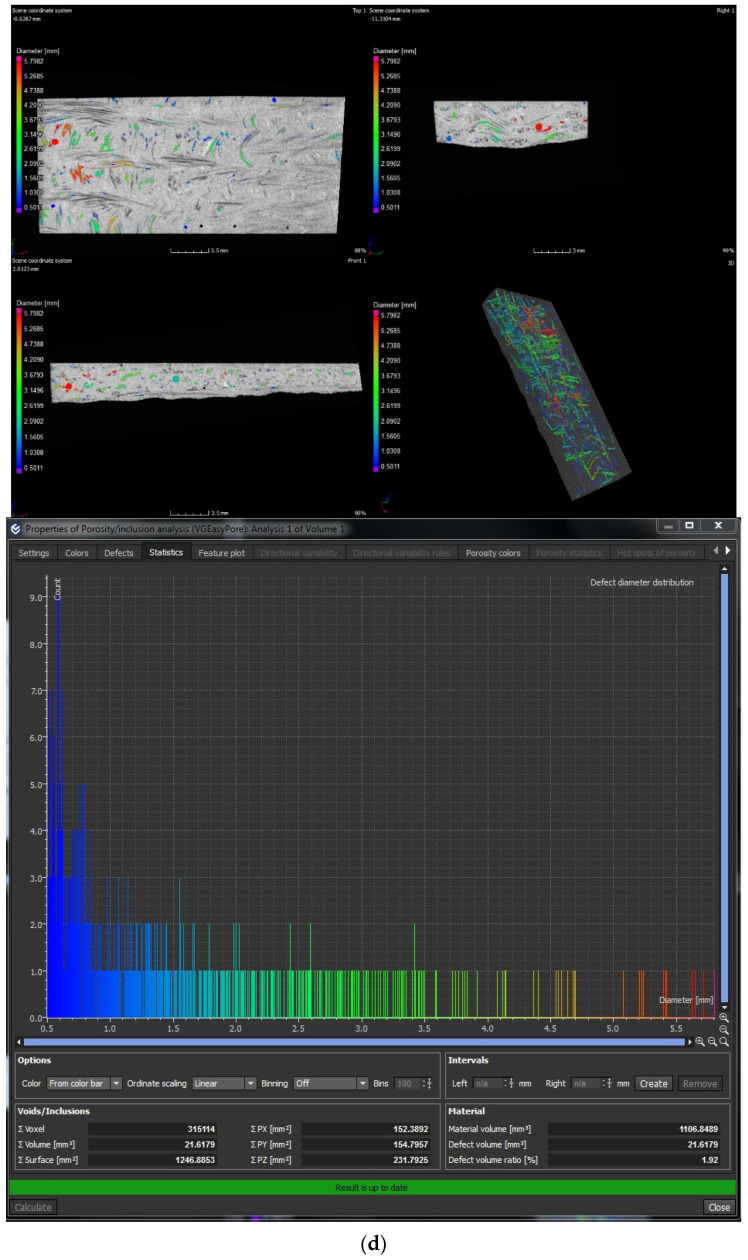
Tomography analysis for the void counts in for (**a**) H4N, (**b**) H4C, (**c**) I4N, and (**d**) I4C.

Figure 3 shows the analysis performed by tomography, and Table 2 presents the number of voids that were present in the laminates.

The henequen laminates present a higher void density in the laminates containing four layers of carbonized fabrics (H4C) and two carbonized layers between the henequen fabrics (HN2CN), referred in Table 1. In contrast, the ixtle laminates have a lower void density than the henequen composites. The ixtle laminates do not show any tendency because the I4N composite presents similar void count than the I4C, and it is higher than the rest of ixtle laminates containing carbonized fabrics.

From a global perspective, it is possible to appreciate that the laminates with four carbonized layers (C4) have the highest number of voids (>1.9%). Although the henequen laminate (N2CN) shows a high volume of defects (2.4%), the rest of the composites had a similar void density that was between 1.2–1.7%.

**Figure 4:**
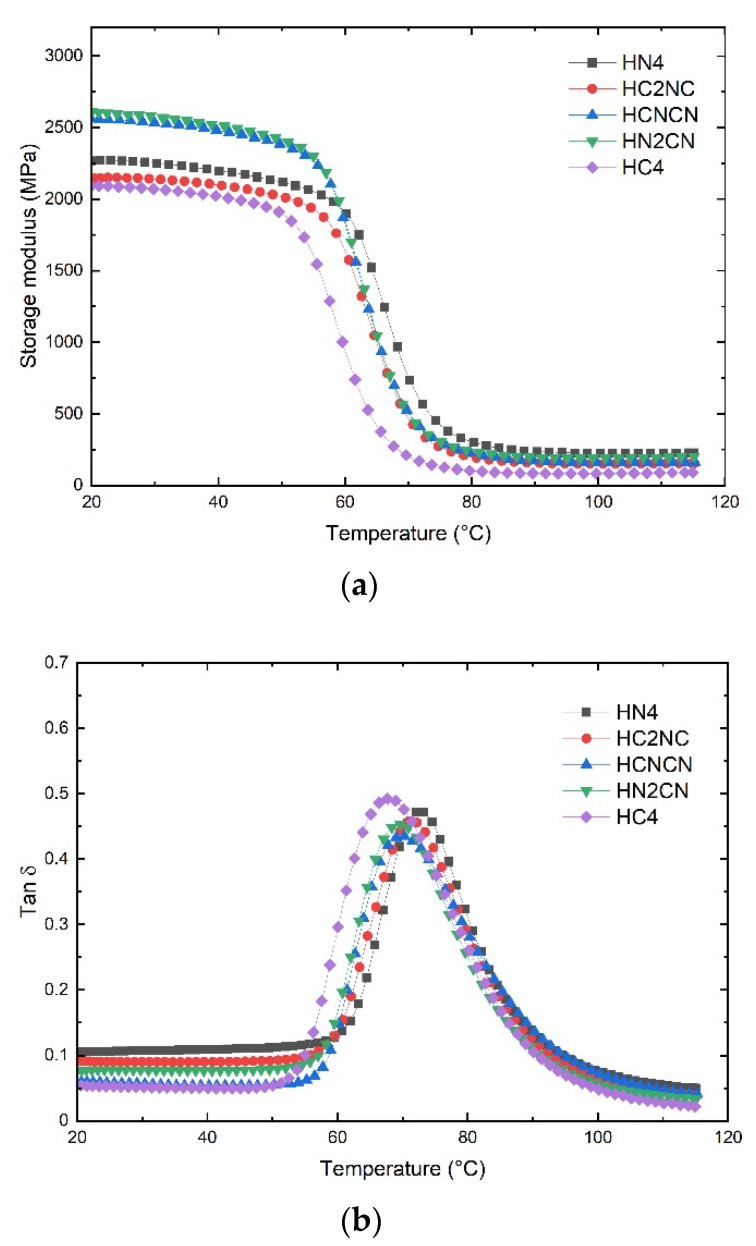
DMA results of non-carbonized and carbonized hybrid henequen laminates: (**a**) storage modulus; (**b**) tan δ.

**Figure 5:**
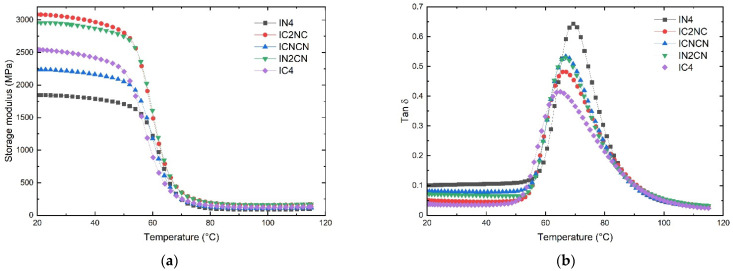
DMA results of non-carbonized and carbonized hybrid ixtle laminates: (**a**) storage modulus; (**b**) tan δ.

Viscoelastic properties such as the storage modulus and the loss factor, or tan δ, were obtained as a function of the temperature from the glassy state to the rubbery plateau. The DMA curves show the viscoelastic response of the henequen and ixtle laminates, which are presented in Figure 4 and Figure 5, respectively.

The dynamic mechanical measurements and viscoelastic behavior in a range of temperatures provide valuable information regarding the structure, morphology, and determination of natural fiber composites [30]. From Figure 4a, it is possible to appreciate that the storage modulus is higher when the base of the laminates consists of non-carbonized henequen fabric.

The HN4 laminate contains four layers of non-carbonized henequen fiber and has an elastic modulus of 2268.5 MPa. This value is similar to that obtained in previous work [1]. When henequen fibers are carbonized, the HC4 laminate reveals an elastic modulus of 2092.1 MPa, representing a reduction of 8.5% concerning the HN4 laminate. This could imply that the carbonized henequen fibers have a lower stiffness due to a porous and brittle composition. On the other hand, the HN2CN and HCNCN laminates show a modulus that is notoriously higher (2612.9 and 2561.1 MPa, respectively) than the rest of the hybrid laminates, including the henequen HN4 laminate (2268.5 MPa). The lowest modulus corresponded to the HC4 composite (2092.1 MPa).

Hybrid henequen laminates are more compact than non-hybrid ones, which results in higher stiffness. Moreover, the structural effect promoted by the plain weave fabric favors the stiffness of the hybrid laminates when the temperature increases; hence, the HN_4_, which was formed with non-carbonized fabrics, shows the highest onset temperature (59.2 °C).

In the rubbery zone (90 °C), the HN4 shows a storage modulus of 237.5 MPa followed by HN2CN (197.6 MPa) and HCNCN (170.1 MPa). The integrity of the henequen fabric, located at the laminate base, significantly impacted the modulus within the rubbery region.

The glass transition temperature (*T_g_)* of laminate composites is commonly obtained from the Tan δ peak in the glass transition region, where the material changes from rigid to more elastic. It is associated with the intermolecular movement of polymer chains [31,32]. Figure 4b shows the effect of the presence of carbonized fibers on the *T_g_* of the henequen laminates. The HN4 peak moves towards the highest temperature (72.8 °C), while the HC4 peak moves to the lowest (67.9 °C). The peaks of the hybrid laminates show practically no relevant changes.

In the case of the HN4 laminate, the henequen fiber fabrics restrict the movement of the polymer chains. However, by replacing these fabrics with carbonized fibers, the structural properties of the laminate decrease, and they do not hinder molecular mobility as much. Therefore, the higher the content of carbonized fiber layers, the lower the resistance to molecular mobility.

Mauricio Torres et al. [1] evaluated the viscoelastic behavior of henequen and ixtle laminates reinforced with ZnO nanoparticles. The authors determined that the henequen and ixtle laminates had a storage modulus of 2260 and 1830 MPa, respectively, and both of those values are similar to those obtained in this work. The authors attributed the differences in the mechanical properties between the two laminates to higher compatibility between EVO bio-based resin and henequen fibers.

From Figure 5a, the IC4 laminate, which contained four layers of carbonized ixtle fiber, shows a storage modulus of 2541.4 MPa, 37.8% greater than the IN4 laminate (1843.9 MPa) and 12% greater than the HN4 laminate (2268.5 MPa).

Mauricio Torres et al. [2] evaluated the tensile mechanical behavior of henequen and ixtle fibers. The authors found that ixtle fibers have an elastic modulus of 26.5 GPa, which is 100.5% stiffer than that of henequen fibers (12.9 GPa). Therefore, the authors attributed the maceration process of the ixtle fibers as one of the causes of the low interaction with the EVO resin, which affected the mechanical properties of the ixtle laminates considerably.

The maceration process leaves a waxy coating on the ixtle fiber so that it can be woven. When ixtle fibers are carbonized, the waxy coating is removed, and the fibers maintain their rigid structure, which enhances the mechanical properties of IC4 and their hybrid laminates with respect to the henequen composites.

Contrary to the results obtained with the henequen laminates, the ixtle hybrid laminates did not show a clear trend since IC2NC showed the highest storage modulus (3082.5 MPa) followed by IN2CN (2954.9 MPa), and the non-carbonized ixtle laminate IN4 turned out to be the least rigid material. Furthermore, most hybrid ixtle laminates are stiffer than HN4, which could also be attributed to the high packing of the stacked layers during the VARI process.

The above should indicate that the manufacturing process of hybrid carbonized and non-carbonized plant fiber systems could influence the mechanical properties of the composites, especially considering the glassy region. In this sense, the carbonized plant fibers should act as an effective filler to manufacture lightweight ecological composites.

On the other hand, Figure 5b shows that the *T_g_* peak corresponding to IN4 shifts towards a higher temperature. Therefore, non-carbonized ixtle fabrics impede molecular movement more markedly than hybrid and carbonized ixtle composites. Several authors agree that the mechanical damping coefficient is the relationship between the loss modulus and the storage modulus and is related to the degree of molecular mobility in the polymeric material [33,34,35,36], where natural fibers have an intrinsic relaxation behavior that has a primary role in the stress relaxation of laminated composites. Therefore, the relaxation of the reinforcing fiber used during the viscoelastic test should be studied in detail [30].

Thermal shock can promote a continuous strain rate that results in the failure of the crosslinks between the fibers and the polymer and the creation of a stress concentration at the interface [37]. The stress concentration in the composite structure reduces the maximum load bearing capacity of composites and limits the stress transition between the fibers and the matrix, creating microcracks at the interface, which leads to delamination.

**Figure 6:**
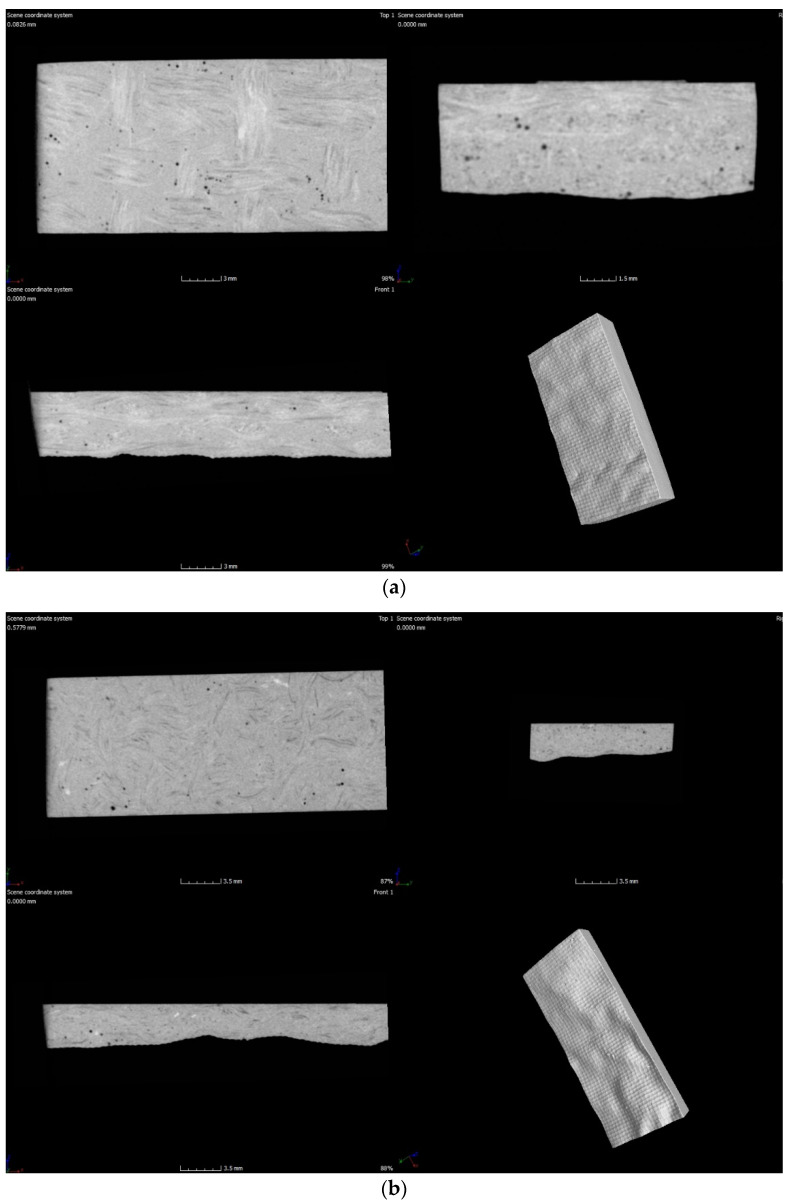

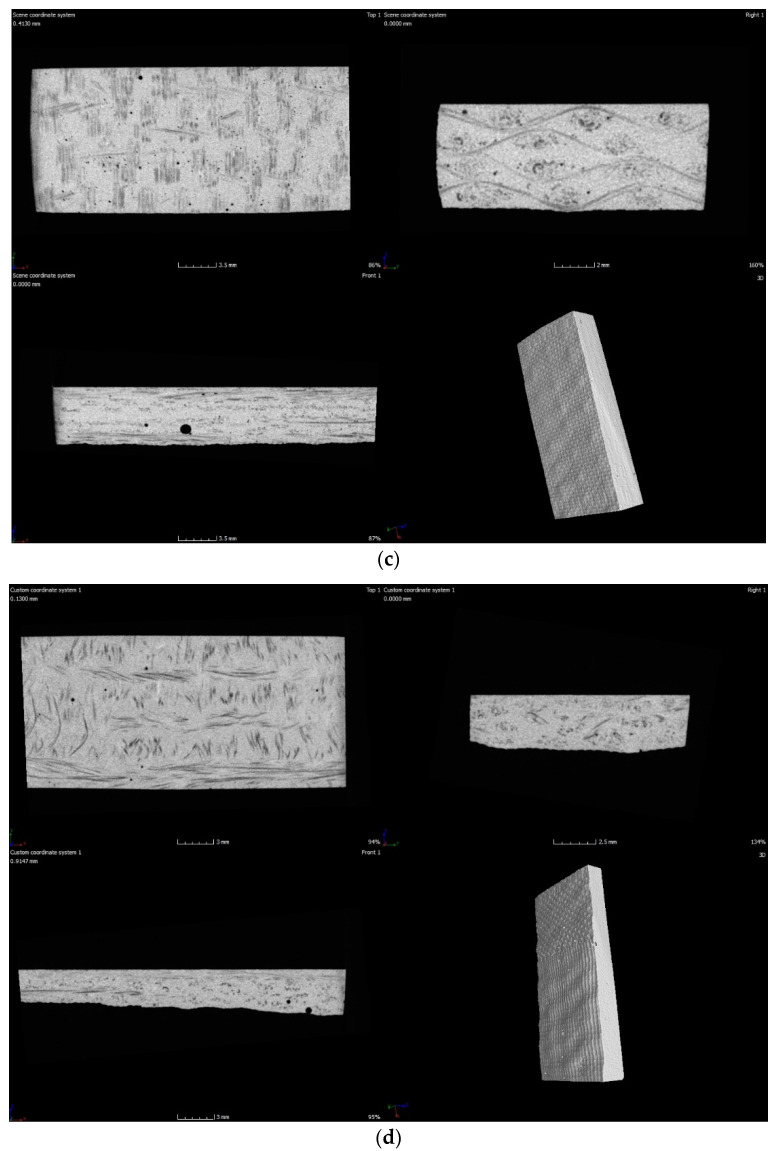
Tomography pictures corresponding to DMA specimens after thermal shock cycling for (**a**) H4N, (**b**) H4C, (**c**) I4N, (**d**) I4C.

DMA specimens were analyzed using an X-ray CT scan before and after the thermal shock cycling. Figure 6 shows that the thermal shock approach did not influence the structural integrity of the DMA specimens because of the absence of the formation of microcracks.

**Figure 7:**
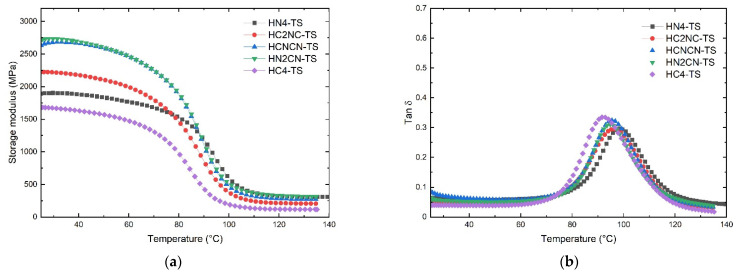
DMA results of non-carbonized and carbonized hybrid henequen laminates after thermal shock cycling: (**a**) storage modulus; (**b**) tan δ.

Figure 7 shows the DMA curves corresponding to the henequen laminates after the thermal shock cycling, and Table 3 lists the viscoelastic parameters (storage modulus and glass transition from Tan δ).

It is possible to appreciate the significant change in the shape of the curve recorded for the storage modulus (Figure 7a). The storage modulus decreased gradually with the temperature within the glassy region for laminates containing carbonized henequen fibers. In contrast, the storage modulus for the HN4 is better defined until the T onset.

The storage modulus increased slightly in the henequen hybrid laminates after thermal shock. In contrast, the storage modulus for the non-hybrid laminates (HN4-TS) and carbonized composites (HC4-TS) decreased markedly to 16.5% and 19.7%, respectively.

Regarding the Tan δ, the thermal shock cycling of the laminates promoted the displacement of the peaks towards higher temperatures than the non-thermal shocked laminates (Figure 7b). The laminate containing henequen fabrics (HN4) showed the highest *T_g_*; meanwhile, the laminate only containing carbonized fibers (HC4) has the lowest *T_g_*.

**Figure 8:**
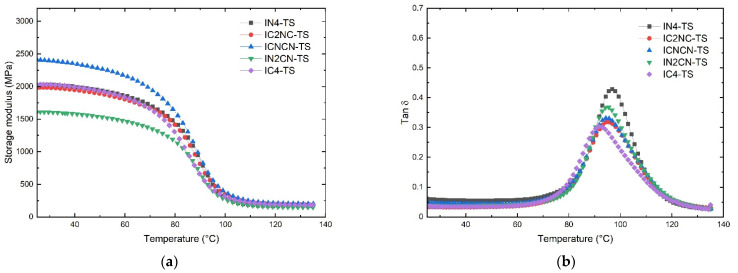
DMA results of non-carbonized and carbonized hybrid ixtle laminates after thermal shock cycling: (**a**) storage modulus; (**b**) tan δ.

On the other hand, the ixtle laminates subjected to the thermal shock cycle showed a behavior similar to the henequen laminates discussed above, with diffusive glassy region until T onset (Figure 8). According to Table 3, the ICNCN-TS had the highest modulus, while the IN2CN-TS showed the lowest modulus. Additionally, tan δ peaks shift to higher temperatures, the IN4-TS displays the highest *T_g_* (96.8 °C), and IC4 displays the lowest (91.9 °C).

The increase of the storage modulus after the thermal shock cycling could be related to the post-curing process promoted by the thermal cycling process. Some authors [37] have observed that the post-curing effect improves the interface quality of composites by increasing crosslinks. They detected two dominant effects during the thermal shock process: First, post-curing occurs with low thermal shock cycles and increases stiffness, contact force, and absorbed energy. Second, debonding appears to be due to the mismatch of the thermal expansion constituent coefficient caused by increasing cycles. In this work, the post-curing process increased the crosslinking effect, which is evident for the increase of *T_g_*.

## 4. Conclusions

The viability of implanting a natural fiber carbonization process to develop laminated composites directed towards integrating commercial products implies knowing the resulting mechanical properties, process costs, and manufacturing process. In terms of the process, it is evident that the carbonization temperatures of natural fibers are much lower than those of fossil fibers. Furthermore, the carbonization process of carbon fiber is continuous, whereas natural carbon fiber must be produced in batches due to the lack of flexibility of carbonized plant fibers. This work estimates that the approximate cost of a 20 × 20 cm four-ply carbonized natural laminate is USD 4, although more complex estimates are required for an industrial process, which is currently being developed in collaboration with other teams.

The FTIR spectrum revealed characteristic bands of styrene instead of organic compounds, which means that the experimental carbonization procedure is adequate to carbonize the agave fabrics.

Tomography scans revealed the presence of microvoids in the laminates. The carbonized laminates of henequen and ixtle presented a higher void density.

The viscoelastic properties revealed that some hybrid laminates showed a higher modulus than the henequen laminate, which was attributed to the higher fabric compaction. The non-carbonized layer of the henequen fabric located in the base of the laminates, i.e., layer number 4, restricted the glassy region’s molecular movement and improved the stiffness of the henequen system. On the contrary, the ixtle systems did not show any such tendency.

The thermal shock procedure promoted an increased storage modulus in the hybrid henequen laminates, but the stiffness decreased in the henequen and carbonized laminates. Once again, the ixtle systems did not display any such tendency. Moreover, thermal shock cycling promoted the post-curing process for all of the laminates evaluated in this work, which was evident, especially considering the notorious increase of the glass transition temperature.

The use of carbonized plant fiber fabrics as a reinforcement for bio-laminates is a vast field of research. This carbonized waste material is much less dense than synthetic fillers, leading to lightweight composites. The results obtained in this work confirm the effective use of carbonized fibers as composite fillers. The research team is currently developing additional research work that includes thermal stability and visual observations through the use of scanning electron microscope (SEM).

## Figures and Tables

**Table 1:** Stacking sequence and configuration of hybrid laminates.

Id	Layer 1	Layer 2	Layer 3	Layer 4	Configuration
4H	H	H	H	H	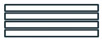
2HC2H	C	H	H	C	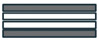
HCNCN	C	N	C	N	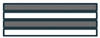
HN2CN	N	C	C	N	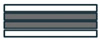
H4C	C	C	C	C	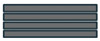
I4N	I	I	I	I	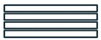
IC2NC	C	I	I	C	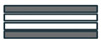
ICNCN	C	I	C	I	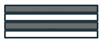
IN2CN	I	C	C	I	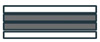
I4C	C	C	C	C	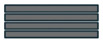

NOTE: Ixtle (I), Henequén (H), Carbonized (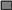 C), non-carbonized (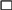 N).

**Table 2:** Porosity volume ratio.

Laminate	Henequen Fiber	Ixtle Fiber
N4	1.3	1.7
C2NC	1.4	1.3
CNCN	1.3	1.2
N2CN	2.4	1.4
C4	2.3	1.9

**Table 3:** Viscoelastic parameters of henequen and ixtle hybrid laminates before and after thermal shock (TS) cycling.

Laminate	Storage Modulus (MPa)				Tg (°C)			
	H	H-TS	I	I-TS	H	H-TS	I	I-TS
N4	2268.5	1895.9	1843.9	2023.9	72.7	98.3	69.4	96.78
C2NC	2138.9	2223.9	3082.5	1982.6	71.4	96.2	66.3	95.1
CNCN	2561.3	2654.3	2238.8	2408.9	69.7	95.4	66.8	94.8
N2CN	2611.5	2730.7	2954.9	1609.7	69.6	94.8	66.5	94.9
C4	2092.1	1680.5	2541.4	2025.3	67.9	92.1	64.7	91.9

## Data Availability

This study did not report any data.

## References

[1] Torres M., Rodriguez V.R., Alcantara P.I., Franco-Urquiza E. (2020). Mechanical properties and fracture behaviour of agave fibers bio-based epoxy laminates reinforced with zinc oxide. J. Ind. Text..

[2] Torres-Arellano M., Renteria-Rodríguez V., Franco-Urquiza E. (2020). Mechanical Properties of Natural-Fiber-Reinforced Biobased Epoxy Resins Manufactured by Resin Infusion Process. Polymers.

[3] Franco-Urquiza E.A., Rentería-Rodríguez A.V. (2021). Effect of nanoparticles on the mechanical properties of kenaf fiber-reinforced bio-based epoxy resin. Text. Res. J..

[4] Malburet S., Di Mauro C., Noè C., Mija A., Sangermano M., Graillot A. (2020). Sustainable access to fully biobased epoxidized vegetable oil thermoset materials prepared by thermal or UV-cationic processes. RSC Adv..

[5] Mustapha R., Rahmat A.R., Majid R.A., Mustapha S.N.H. (2019). Vegetable oil-based epoxy resins and their composites with bio-based hardener: A short review. Polym. Technol. Mater..

[6] Saurabh T., Patnaik M., Bhagt S.L., Renge V.C. (2011). Epoxidation of vegetable oils: A review. Int. J. Adv. Eng. Technol..

[7] Ng F., Couture G., Philippe C., Boutevin B., Caillol S. (2017). Bio-Based Aromatic Epoxy Monomers for Thermoset Materials. Molecules.

[8] Nikafshar S., Zabihi O., Hamidi S., Moradi Y., Barzegar S., Ahmadi M., Naebe M. (2017). A renewable bio-based epoxy resin with improved mechanical performance that can compete with DGEBA. RSC Adv..

[9] Ray D., Sain S. (2017). Thermosetting bioresins as matrix for biocomposites. Biocomposites for High-Performance Applications.

[10] Auvergne R., Caillol S., David G., Boutevin B., Pascault J.-P.P. (2014). Biobased Thermosetting Epoxy: Present and Future.

[11] Miyagawa H., Jurek R.J., Mohanty A.K., Misra M., Drzal L.T. (2006). Biobased epoxy/clay nanocomposites as a new matrix for CFRP. Compos. Part A Appl. Sci. Manuf..

[12] Vasquez-Zacarias L., Ponce-Peña P., Pérez-López T., Franco-Urquiza E.A., Ramirez-Galicia G., Poisot M. (2018). Hybrid Cellulose-Silica Materials from Renewable Secondary Raw Resources: An Eco-friendly Method. Glob. Chall..

[13] Thyavihalli Girijappa Y.G., Mavinkere Rangappa S., Parameswaranpillai J., Siengchin S. (2019). Natural Fibers as Sustainable and Renewable Resource for Development of Eco-Friendly Composites: A Comprehensive Review. Front. Mater..

[14] Shekar H.S.S., Ramachandra M. (2018). Green Composites: A Review. Mater. Today Proc..

[15] Parbin S., Waghmare N.K., Singh S.K., Khan S. (2019). Mechanical properties of natural fiber reinforced epoxy composites: A review. Procedia Comput. Sci..

[16] Akil H.M., Omar M.F., Mazuki A.A.M., Safiee S., Ishak Z.A.M., Abu Bakar A. (2011). Kenaf fiber reinforced composites: A review. Mater. Des..

[17] Pistor V., Soares S.S.D.S.D.S., Ornaghi H.L., Fiorio R., Zattera A.J. (2012). Influence of glass and sisal fibers on the cure kinetics of unsaturated polyester resin. Mater. Res..

[18] Chand N., Fahim M. (2008). Natural fibers and their composites. Woodhead Publishing Series in Composites Science and Engineering.

[19] Ramamoorthy S.K., Skrifvars M., Persson A. (2015). A review of natural fibers used in biocomposites: Plant, animal and regenerated cellulose fibers. Polym. Rev..

[20] Balint T., Chang B.P., Mohanty A.K., Misra M. (2020). Underutilized agricultural co-product as a sustainable biofiller for polyamide 6,6: Effect of carbonization temperature. Molecules.

[21] Vukcevic M., Kalijadis A., Radisic M., Pejic B., Kostic M., Lausevic Z., Lausevic M. (2012). Application of carbonized hemp fibers as a new solid-phase extraction sorbent for analysis of pesticides in water samples. Chem. Eng. J..

[22] Poursorkhabi V., Mohanty A., Misra M. (2015). Characterization of electrospun lignin based carbon fibers. AIP Conf. Proc..

[23] Zhang M., Wang C., Wang H., Jian M., Hao X., Zhang Y. (2017). Carbonized Cotton Fabric for High-Performance Wearable Strain Sensors. Adv. Funct. Mater..

[24] Spörl J.M., Beyer R., Abels F., Cwik T., Müller A., Hermanutz F., Buchmeiser M.R. (2017). Cellulose-Derived Carbon Fibers with Improved Carbon Yield and Mechanical Properties. Macromol. Mater. Eng..

[25] Orue A., Jauregi A., Peña-Rodriguez C., Labidi J., Eceiza A., Arbelaiz A. (2015). The effect of surface modifications on sisal fiber properties and sisal/poly (lactic acid) interface adhesion. Compos. Part B Eng..

[26] Jones R.N., Ramsay D.A., Keir D.S., Dobriner K. (1952). The Intensities of Carbonyl Bands in the Infrared Spectra of Steroids1. J. Am. Chem. Soc..

[27] WU H., FAN S., YUAN X., CHEN L., DENG J. (2013). Fabrication of carbon fibers from jute fibers by pre-oxidation and carbonization. New Carbon Mater..

[28] Hamidi Y.K., Aktas L., Altan M.C. (2004). Formation of microscopic voids in resin transfer molded composites. J. Eng. Mater. Technol. Trans. ASME.

[29] Suzuki Y., Cousins D.S., Dorgan J.R., Stebner A.P., Kappes B.B. (2019). Dual-energy X-ray computed tomography for void detection in fiber-reinforced composites. J. Compos. Mater..

[30] Moriana R., Vilaplana F., Karlsson S., Ribes-Greus A. (2011). Improved thermo-mechanical properties by the addition of natural fibres in starch-based sustainable biocomposites. Compos. Part A Appl. Sci. Manuf..

[31] Gheith M.H., Aziz M.A., Ghori W., Saba N., Asim M., Jawaid M., Alothman O.Y. (2019). Flexural, thermal and dynamic mechanical properties of date palm fibres reinforced epoxy composites. J. Mater. Res. Technol..

[32] Dhar Malingam S., Lin Feng N., Chan K., Subramaniam K., Selamat M., Zakaria K.A. (2018). The Static and Dynamic Mechanical Properties of Kenaf/Glass Fibre Reinforced Hybrid Composites. Mater. Res. Express.

[33] Franco-Urquiza E.A., Cailloux J., Santana O., Maspoch M.L., Velazquez Infante J.C. (2015). The Influence of the Clay Particles on the Mechanical Properties and Fracture Behavior of PLA/o-MMT Composite Films. Adv. Polym. Technol..

[34] Pothan L.A., Cherian B.M., Anandakutty B., Thomas S. (2007). Effect of layering pattern on the water absorption behavior of banana glass hybrid composites. J. Appl. Polym. Sci..

[35] Pothan L.A., George C.N., John M.J., Thomas S. (2009). Dynamic Mechanical and Dielectric Behavior of Banana-Glass Hybrid Fiber Reinforced Polyester Composites. J. Reinf. Plast. Compos..

[36] Priya S.P., Rai S.K. (2006). Mechanical Performance of Biofiber/Glass-reinforced Epoxy Hybrid Composites. J. Ind. Text..

[37] Azimpour-Shishevan F., Akbulut H., Mohtadi-Bonab M.A. (2019). The Effect of Thermal Shock Cycling on Low Velocity Impact Behavior of Carbon Fiber Reinforced Epoxy Composites. J. Dyn. Behav. Mater..

